# Is Alcohol Food?

**Published:** 1874-06

**Authors:** 


### Editorial.

Is Alcohol Food?—Much discussion has been going on for a year past in the medical periodicals, especially of Great Britain, but to a considerable extent in this country also, on the action of alcohol upon the human system, both in health and disease. The question whether alcohol is capable of becoming assimilated with the tissues of the body by chemical changes, so as to be capable of taking rank among nutritive materials, has divided the minds of physiologists to some extent; and the methods of elimination of it from the human system have engaged much thought and careful experiment.

Now that the gigantic evils which follow upon the popular abuse of alcoholic stimulants in their varied forms are engaging the anxious attention and active counter-exertions of the wise, the good, the philanthropic all over our land; now that the patience, the meekness, the long-suffering of the poor defenceless wife, in utter desperation breaks the bonds which have for so many long, weary years pent up within her own bosom the dark, sad record of unrequited love, blasted hopes, crushed aspirations, deep and abiding wrongs, and pours itself forth at the very door of the dram-shop, and at noon-day, in heart-broken supplications to a supernatural power, where resides justice, and mercy, and vengeance; now that our State Legislatures are being aroused to the necessity for vigorous action by petitions pouring in from every quarter, supplicating protection for the people against the baleful tricklings of the poisonous copper worm, the buck-eye, the strychnia, the dead-shot, and demanding that inebriety be branded as lunacy, and its victims be put in durance for their own safety and the public good, it well behooves our profession to give the subject deep and serious attention, and more especially in its physiological, pathological and psycological bearings.

Whilst the physiologists, with their ample means and opportunities of laboratory experiment and study, and the pathologists, with their extended fields of research amongst the victims of alcoholic poisoning in our great charity hospitals, are engaged in working up their respective branches of the great social problem, it is our humble privilege—thanks to the watchfulness of that careful and skilled student of nature, especially *human* nature,

William Earpe, Esq., Bill Arp, so called—to add a single contribution, not unimportant we hope in its bearings upon the psycological branch of the inquiry, illustrative of the expanding influence of alcohol upon the cerebral hemispheres, or rather upon the phenomenon of intellection, the resultant of cerebral action under alcoholic influence.

Case.—Mr. Blank, æt. thirty, farmer, married, of strong, vigorous constitution, and in his usual health, visited the Mountain City for the purpose of laying in a small stock of "groceries." Meeting a county official upon the street, he accosted him:

"Adolphus, I learn you intend visiting Texas?" "Yes."

"Well, I have got a brother out there named Ben. If you should chance to meet him, say to him that we are getting along badly here in Georgia. Tell him I have got but six acres in wheat, and it is so badly damaged I don't think it will make anything. Tell him sister Sallie married a few weeks ago, a trifling fellow who is of no account. Tell him, if he can, do send us a little money to help us through the year."

Having circulated a little among the "groceries," and imbibed a few doses of the spiritus frumenti, he again encounters the official:

"Say! Dolphus, they tell me you are going to Texas?" "Yes."

"Well, you'll be apt to see brother Ben, and if you do, tell him I have got twelve acres in wheat, and its very promising. It'll make twenty-five bushels to the acre. Tell him sister Sal's married to a mighty clever fellow. He ha'n't got much, but he's industrious and he'll soon get up in the world. Tell him we are all doing first rate."

Another round of inspection in the "groceries," and the parties again meet:

"Say there, Dolph! 'tell me you're goin' Texas?" "Yes."

"Well, you'll be *sure* to meet brother Ben; *he* lives in Texas. Tell him for me, Dolph, that we are all doin' splendid. Tell him I've got thirty acres of the finest wheat he ever did see; it'll make fifty bushels to the acre. Tell him Sal married the cleverest fellow in Chattooga county, and he's rich, too. Tell him if he needs any money, why, just let us know, and we'll send him some, *sure.*"

Another, and more arduous, tour of inspection so far exhausted his physical powers, in his mental expansion, as to render his gait reeling and unsteady when the policeman carried him off to the "lock-up." Moved by commendable human sympathy, the kind-hearted official visited him later in the day, and through the grated window of his cell received his final message to his distant relative:

"Say! Dolph, say! 'tell me you're goin' Texas?" "Yes."

"Well, if you *do* see brother Ben, tell him that the rivers is riz, the country's overflowed, and h—'s turned loose!"

